# Simulations of foil-based spin-echo (modulated) small-angle neutron scattering with a sample using *McStas*

**DOI:** 10.1107/S1600576720015496

**Published:** 2021-02-01

**Authors:** Wim G. Bouwman, Erik B. Knudsen, Linda Udby, Peter Willendrup

**Affiliations:** a Delft University of Technology, The Netherlands; b Technical University of Denmark, Denmark; c University of Copenhagen, Denmark

**Keywords:** small-angle scattering, spin-echo methods, polarized neutrons, Monte Carlo simulations

## Abstract

For the further development of spin-echo techniques to label elastic scattering it is necessary to perform simulations of the Larmor precession of neutron spins in a magnetic field. The details of some of these techniques as implemented at the reactor in Delft are simulated. First, the workings of the magnetized foil flipper are simulated. A full virtual spin-echo small-angle neutron scattering instrument is built and tested without and with a realistic scattering sample. It is essential for these simulations to have a simulated sample that also describes the transmitted beam of unscattered neutrons, which usually is not implemented for the simulation of conventional small-angle neutron scattering (SANS) instruments. Finally, the workings of a spin-echo modulated small-angle neutron scattering (SEMSANS) instrument are simulated. The simulations are in good agreement with theory and experiments. This setup can be extended to include realistic magnetic field distributions to fully predict the features of future Larmor labelling elastic-scattering instruments. Configurations can now be simulated for more complicated combinations of SANS with SEMSANS.

## Abstract 2

Simulations of a foil-based spin-echo small-angle neutron scattering instrument are performed and agree with analytical calculations. The simulation tools for polarization manipulation can be used to optimally design new spin-echo instruments.

## 1. Introduction   

To obtain a high resolution (*e.g.* smaller scattering vectors or smaller energy transfer) with conventional scattering methods, normally the beam size, divergence or wavelength bandwidth have to be reduced, with a corresponding loss in flux. For neutron scattering this can limit the highest resolution that can be practically achieved with an acceptable neutron count rate. Spin-echo methods can circumvent this intensity problem (Mezei *et al.*, 2002 ▸). These polarized neutron methods use the Larmor precession in magnetic fields to measure the change in energy or direction of scattered neutrons. By tilting the interfaces of the precession regions with respect to the optical axis of the neutron beam, the elastic scattering can be measured with a high resolution (Rekveldt *et al.*, 2003 ▸; Bouwman *et al.*, 2008 ▸). Spin-echo small-angle neutron scattering (SESANS) is one of these techniques (Rekveldt *et al.*, 2005 ▸). SESANS can measure structures with length scales from 10 nm up to 20 µm corresponding to scattering vector transfers from 3 × 10^−5^ to 6 × 10^−2^ Å^−1^.

A more recent variant of this technique is spin-echo modulated small-angle neutron scattering (SEMSANS) (Gähler, 2006 ▸; Bouwman *et al.*, 2009 ▸; Sales *et al.*, 2015 ▸), in which all polarization manipulations occur before the sample. This makes it also possible to combine SEMSANS and small-angle neutron scattering (SANS) (Bouwman *et al.*, 2011 ▸).

It is challenging to understand the details on how these techniques work, the connection to conventional SANS (Krouglov *et al.*, 2003 ▸; Andersson *et al.*, 2008 ▸; Kohlbrecher & Studer, 2017 ▸) and how to reduce the data (Sales *et al.*, 2017 ▸). Specifically, the data analysis for time-of-flight SE(M)SANS measurements is challenging because of finite size acceptances and scattering powers that are dependent on the wavelength (Li *et al.*, 2019 ▸).

### 1.1. *McStas*   

For the design and optimization of neutron instrumentation, Monte Carlo simulations play an important role. Mathematical models of the neutron sources and components describe the neutron paths through the instruments to the detectors. Originally intended to perform simulations for a new triple-axis spectrometer at Risø National Laboratory, the *McStas* (Lefmann & Nielsen, 1999 ▸; Willendrup & Lefmann, 2019 ▸; *McStas*, 1998 ▸) Monte Carlo ray-tracing code was initiated in Denmark in 1998. Since then, the use of the software has grown to encompass all types of instrumentation for neutron scattering, and it is very widely used at neutron scattering facilities and universities for simulating the expected outcome of experiments and behaviour of neutron instruments. *McStas* is an open-source (GPLv2, 1991 ▸) software intended for instrument scientists at neutron facilities and serves as a general simulation framework, into which their own developments and contributions can be adapted.

*McStas* has included simple means for simulation of neutron polarization since its early days, but has in this respect been practically useful since version 1.10 from 4 December 2006. Since then, a number of relevant components needed for the simulation of neutron precession and other spin-related physics applications have been added, including neutron polarizers, spin flippers and precession fields. *McStas* uses a modified version of the Seeger–Daemen algorithm (Seeger & Daemen, 2001 ▸) to simulate the Larmor precession in an inhomogeneous magnetic field with high efficiency.

These underlying *McStas* capabilities of computing Larmor precession in magnetic fields have been described and validated by Knudsen *et al.* (2014 ▸), and a basic version of simulated SESANS was shown by Knudsen *et al.* (2011 ▸). Several versions of SESANS and even a SEMSANS experiment (Sales, 2014 ▸) have been simulated, but never with a realistic sample.

Another package for polarized neutron Monte Carlo simulations is *VITESS* (Wechsler *et al.*, 2000 ▸). *VITESS* has simulated radio frequency flippers and spin-echo spectrometers (Manoshin *et al.*, 2016 ▸). With this package a variant of SESANS has been analysed without a sample. However, realistic SANS samples are still missing in *VITESS*, so for the study at hand we have utilized full *McStas* SESANS and SEMSANS instrument descriptions combined with a realistic sample to give more insight into the techniques.

In this article we simulate first all components needed to construct a SESANS or SEMSANS setup. Then we simulate a minimalized SESANS instrument using idealized magnetic fields and a realistic sample for the first time. The simulations on the sample by itself already give interesting perspectives on normal SANS and on SESANS. The complete simulations give insight into the principles of SESANS and SEMSANS. These tools will make it possible to design and optimize future SESANS instruments.

## 2. Simulations   

### 2.1. Hardware and software   

*McStas* version 2.5 was used for the simulations. These simulations were performed on a conventional laptop with an Intel Core i5-8350U quad-core 1.7 GHz CPU. The calculations for a single data point of a SESANS simulation took 13 s for 10^6^ neutrons. A full simulation of a complete SESANS measurement took 15 min. A simulation of a single SEMSANS measurement with 10^8^ neutrons took 10 min.

### 2.2. Source   

A simple source is used to represent the wavelength distribution used in Delft for SESANS, as selected by the monochromator. The wavelength has a peak at λ = 2.165 Å and half-spread of dλ = 0.02 Å, corresponding to the mosaic spread of the pyrolytic graphite monochromator. We used a beam size of 10 ×  10 mm in all of the simulations with the foil flippers, which is nearly the same as that used with the real instrument. All other components were made much larger to intercept all the neutrons. An arbitrary intensity for the source was used, since we are only looking at the principles of the techniques. For most simulations 10^6^ simulated neutrons are used for each data point in a graph. However, for the SEMSANS simulations 10^8^ simulated neutrons per setting are used to get sufficient counts in each detector pixel. The longer simulation times do not imply that SESANS is more efficient in the use of neutrons than SEMSANS, but merely reflect minor differences between the inner workings of the simulations in the two cases. A larger number of neutrons is required for the SEMSANS simulations because the signal is counted on a detector with 1001 pixels. The graph is easier to interpret if the statistical noise is low. For a real experiment with full analysis the larger number of neutrons would not be necessary, since each neutron still carries approximately the same amount of information.

### 2.3. SANS sample   

SESANS measures both the scattered and the transmitted beam, in contrast to SANS where only a part of the scattered beam outside the beamstop is measured. The most intense parts of the scattering curve contribute the most to the final signal. It is therefore efficient if the simulation sample has a distribution of the simulated neutrons mimicking the real scattered and transmitted contributions. The standard SANS samples from *McStas* consider only the scattered beam with an equal distribution in the wavevector transfer *Q*, which makes them inappropriate for these simulations. Henrich Frielinghaus has contributed a SANS benchmarking sample (SANS_benchmark2) that simulates both the scattered neutrons including multiple scattering and the transmitted neutrons (*McStas*, 1998 ▸). In all of the presented calculations in this article the multiple scattering is included in the calculations. A realistic feature is that the simulated scattered neutrons are equally distributed on a logarithmic *Q* scale, which means that most simulated neutrons go to where the high intensity is, in contrast to the earlier *McStas* SANS samples with a flat distribution of simulated neutrons. We adapted this component to simulate the scattering of solid spheres for the length scales that match the typical range of SESANS instruments.

The sample consists of solid spheres with a radius *R* = 1.0 µm, a scattering length density contrast of Δρ = 6.0 × 10^10^ cm^−2^, a volume fraction ϕ = 0.015 and a sample thickness of *t* = 1 mm. The radius of 1.0 µm is well within the range of most SESANS instruments and too large for most SANS instruments. The sample is considered to be dilute and no structure factor is taken into account.

An important parameter in SESANS (and ultra-small-angle neutron scattering) is the scattering power τ (Rehm *et al.*, 2013 ▸), which is the average number of scattering events occurring for a neutron while traversing the sample or the total scattering probability. The scattering power is the product of the sample thickness and the macroscopic cross section (*i.e.* the total small-angle scattering cross section per unit volume). With these parameters the scattering power τ will have a value of (Krouglov *et al.*, 2003 ▸; Šaroun, 2000 ▸) 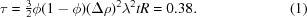
In conventional SANS, scientists typically try to have a scattering power smaller than 0.01, to avoid multiple scattering. In SESANS a scattering power in the range between 0.1 and 0.8 is the optimum for the signal-to-noise ratio (Van Heijkamp, 2011 ▸; Rehm *et al.*, 2013 ▸), since multiple scattering is easily taken into account.

**Figure 1:**
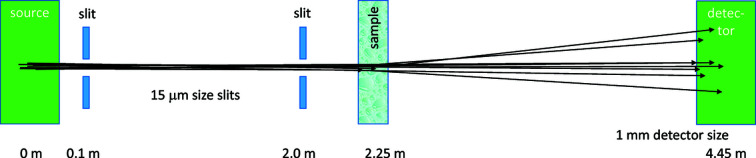
Schematic drawing of the *McStas* components used to test the sphere sample. Basically, it is a SANS instrument. Distances, sizes and angles are not to scale. The same sample and distances were used for the SESANS simulations, but with a much larger beam cross section.

To test the sample a simple SANS implementation is simulated, with the typical distances and wavelength used for the SESANS instrument as sketched in Fig. 1 ▸. To obtain a high enough resolution to measure the small-angle scattering pattern, diaphragm sizes of 15 µm are used. In SESANS typical beam sizes are 15 mm, so a factor of 10^3^ larger than was needed in this simulation. This means that the accepted beam cross section and the accepted divergence are both 10^6^ times as large, leading to the total accepted neutron flux being a factor of 10^12^ higher for the simulated SESANS. This is not a fair comparison, since with SESANS only the scattering in one dimension is analysed, and the setup has only been optimized for SESANS, but it shows that SESANS does use the neutrons more efficiently than SANS (Bouwman *et al.*, 2004 ▸). A position-sensitive detector with a radius of 1 mm was enough to capture the scattering pattern. The pixel size was 8 µm, below the resolution of the diaphragms.

**Figure 2:**
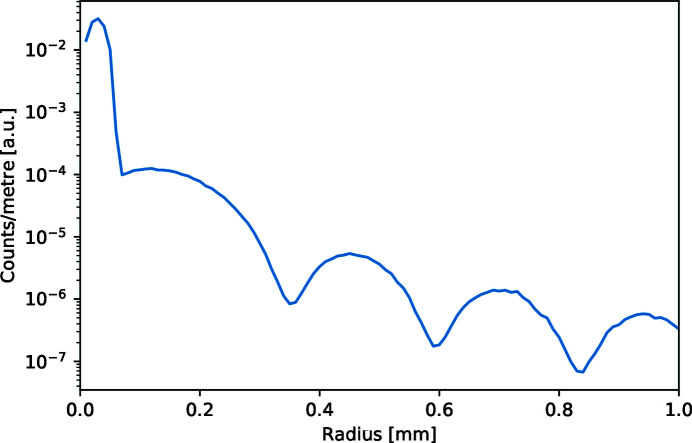
Radially integrated intensity as a function of distance from the centre of the direct beam. The error bars of the simulation are smaller than the linewidth. The integral of the simulated counts is directly proportional to the scattered intensity. To observe with a SANS setup the small-angle scattering of particles with a radius of 1 µm, a narrow beam and a high-resolution position-sensitive detector are needed. The maximum wavevector in this graph corresponds to 1.32 × 10^−3^ Å^−1^.

The number of detected small-angle-scattered neutrons is radially integrated, as shown in Fig. 2 ▸. The advantage of radial integration above conventional radial averaging in this figure is that the integral of the plotted function is directly proportional to the scattered intensity over the corresponding angle. Owing to the realistic sample simulation, this intensity also includes the unscattered transmitted beam component at a radius below 0.07 mm. We have deliberately chosen to present the data in experimental units to show the high resolution that will be needed to measure particles this large with a short instrument. The corresponding wavevector range can be calculated directly from the geometry and the wavelength used to be from 0 to 1.32 × 10^−3^ Å^−1^. The intensity does not peak at radius zero, since that area is not as large as at a larger radius. At a distance from the centre greater than 0.07 mm the typical SANS pattern of solid spheres with the interference peaks is observed. The depth of the valleys is determined by the resolution, because of the finite size of the diaphragms.

### 2.4. Magnetized foil flipper   

**Figure 3:**
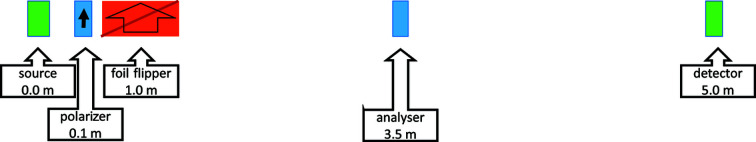
Schematic drawing of the configuration to test a single foil flipper. Distances, sizes and angles are not to scale. To test the functioning of the foil flipper, the polarizer sets the neutron polarization in the positive vertical direction, the non-precession mode. In the SE(M)SANS modes the polarizer sets the neutron polarization in the horizontal direction. The arrow in the foil flipper indicates the direction of the magnetic field. The analyser can be oriented in the positive or negative direction to measure either the up or down intensity.

The preferred method for the thermal beam at the reactor in Delft (Rekveldt *et al.*, 2005 ▸) is to have tilted precession interfaces, achieved by means of tilted magnetized foil flippers (Kraan *et al.*, 2003 ▸) in a large homogeneous tunable magnetic field. The normal of the foils is tilted from the vertical axis by a small angle over the horizontal axis perpendicular to the optical axis of the instrument, as shown schematically in the side view in Fig. 3 ▸. The magnetized foils flip the spin of the neutrons over π rad for a specific wavelength. This π-flip reverses mathematically the sense of rotation of the spin, thus effectively reversing the precession field. The neutrons start to precess while entering the magnetic field in one direction. They are flipped in the foil and precess effectively in the other direction after the magnetic foil. A neutron with a higher path than the optical axis will thus have a longer flight path in the magnetic field before it encounters the foil. A horizontal shift will not make any difference to the length of the neutron path before encountering a foil. The net precession of the polarization in the complete foil flipper and surrounding magnetic field will thus depend on the height of the neutron path through the foil, as is further explained by Kraan *et al.* (2003 ▸).

The change in polarization **P** with time due to the magnetic field **B** is described by 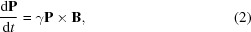
in which γ = 1.83 × 10^8^ s^−1^ T^−1^ is the gyromagnetic ratio of the neutron. As a result the polarization of a polarized beam precesses around the magnetic field with a frequency 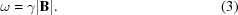
When the polarization vector is parallel to the magnetic field no precession will occur. Further details on the simulation of the precession are given by Knudsen *et al.* (2014 ▸).

The magnetized foils are modelled as mathematical planes, canted by an angle θ, inside a region of constant magnetic field with defined rectangular dimensions. The precession angle ϕ due to this plane is the precession within the film, calculated as (Kraan *et al.*, 2003 ▸) 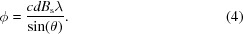
Here, *c* = 4.63 × 10^14^ T^−1^m^−2^ is the Larmor precession constant, calculated via *c* = γ*m*_n_/*h* with *m*_n_ the mass of the neutron and *h* Planck’s constant, *B*_s_ = 1.0 T is the saturation magnetization of the magnetic foil, θ = 0.0960 rad is its tilt angle and *d* = 3.0 µm is its thickness. The rotation of the polarization vector is taken over the optical axis of the setup, to mimic the *x* coils in the foil flippers which set the local magnetic field perpendicular to the foil plane. In real SESANS, the *x* coils add a component to the applied magnetic field in the direction of the neutron path to turn the plane in which the neutron spins are oriented in the field of the magnetized foil to obtain a perfect π-flip. For further details and explanation we refer to the article by Kraan *et al.* (2003 ▸).

**Figure 4:**
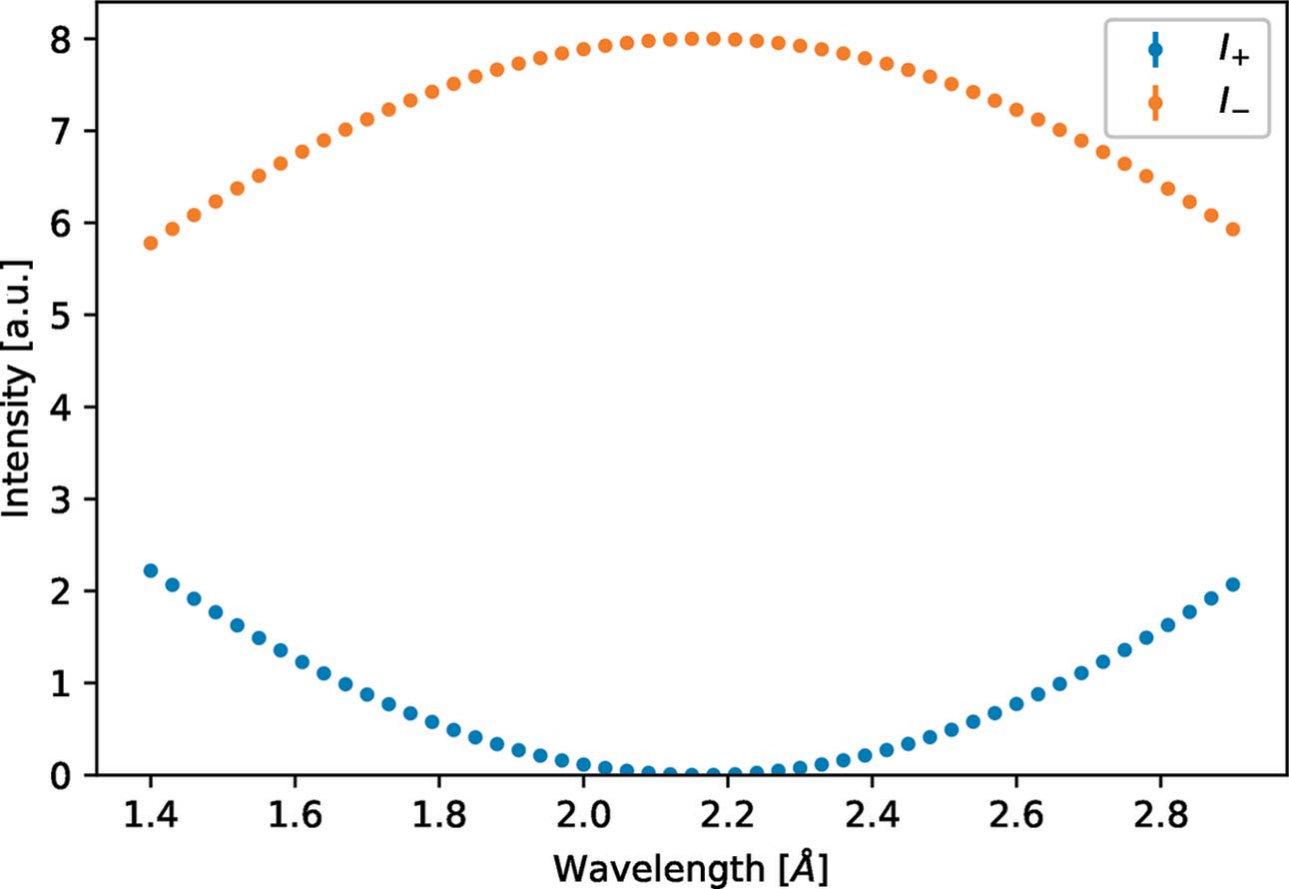
Simulated intensity as a function of wavelength of a polarized neutron beam after passing a single foil flipper in the non-precession mode. The error in the simulation was less than the thickness of the markers. *I*_+_ and *I*_−_ are the intensities measured with the analyser oriented in the up and down orientations.

The foil flipper is tested with a setup as sketched in Fig. 3 ▸ in the non-precession mode. The positions of the analyser and detector are not important, but they match approximately the positions used later on for the SESANS and SEMSANS simulations. The polarizer was set to transmit neutrons with their spin in the positive vertical direction. As a result there should be no precession before and after the foil. For the right wavelength there should occur a π-flip of the polarization, as can be seen in Fig. 4 ▸. The wavelength of the source was scanned for this simulation. The analyser was set to transmit the neutrons with their spin in either the positive or negative vertical direction. The plus transmission goes to zero for a wavelength of λ = 2.165 Å, as one would expect for a rotation ϕ = π from equation (4). The simulation is in good agreement with the measurements presented in Fig. 4 of Kraan *et al.* (2003 ▸). The test in the precession mode is described in the following section on SESANS.

## 3. SESANS   

**Figure 5:**
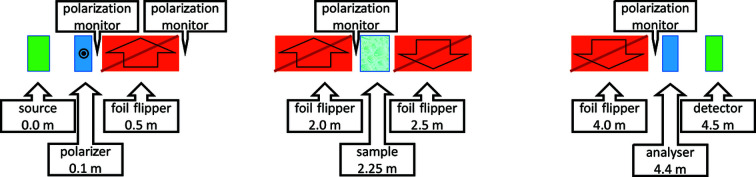
Schematic drawing of the *McStas* SESANS setup. Distances, sizes and angles are not to scale. The arrows in the foil flippers indicate the direction of the magnetic field. The polarizer sets the neutron polarization out of the plane of the drawing. Polarization monitors are positioned at several critical positions in the instrument. The setup was tested without and with the sample.

The simulation setup, as sketched in Fig. 5 ▸, has only the essential elements to simulate the Larmor labelling and the scattering processes occurring. For these simulations the polarizer and analyser transmit only neutrons with a spin in the horizontal direction, perpendicular to the neutron beam. We omitted for simplicity the π/2 flippers (Kraan *et al.*, 1991 ▸) that start and stop the precession in the real instrument (Knudsen *et al.*, 2011 ▸). The distances are similar to those in the SESANS setup in Delft (Rekveldt *et al.*, 2005 ▸). The beam height and width are both 10 mm.

**Figure 6:**
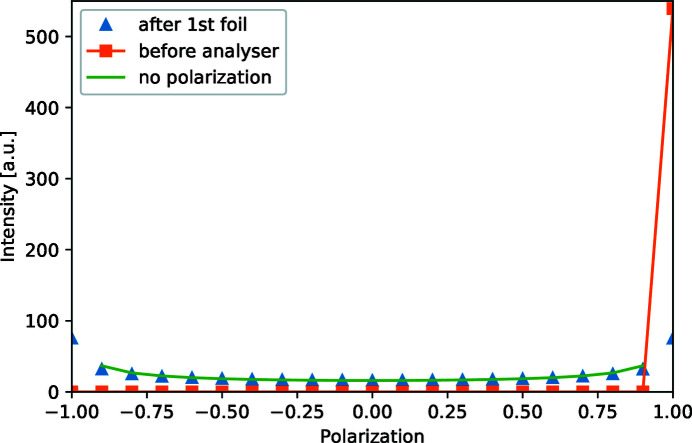
The polarization distribution monitored in several locations of the SESANS simulation. The distributions just after the polarizer and just before the analyser are perfectly overlapping, so only the distribution just before the analyser is plotted in this graph. The distribution after the first and second foil also perfectly overlap, so only the polarization distribution directly after the first foil is plotted. They match with the calculated flat distribution in precession angle of a dephased beam.

The instrument is first tested without a sample with polarization monitors after the polarizer, after the first foil, at the sample position and just before the analyser. The direct output of these four polarization monitors illustrates the spin-echo principle. Just after the polarizer the polarization is unity, as shown in Fig. 6 ▸. Directly after the first foil flipper the polarization of the neutron beam is completely lost, and the same is true at the sample position. The losses of polarization are due to the fact that neutron trajectories have been summed over the full beam width with some contribution from the divergence and wavelength spread. The distribution is higher at the polarizations of −1.0 and 1.0 than in between at a polarization of 0. This is because the distribution is flat in precession angle ϕ. The corresponding monitored projection is the polarization 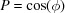. From this one can calculate the distribution of the intensity over the polarization by taking the derivative of the inverse projection with respect to precession angle: 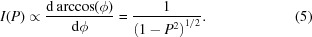
This calculated intensity matches the simulation, except for the points where |*P*| = 1. This mismatch at the ends occurs because in the simulation the polarization is binned, while the calculation of the intensity with equation (5) diverges at |*P*| = 1. The polarization is completely recovered after the fourth foil flipper, before the analyser, as can be seen in Fig. 6 ▸. This perfect spin echo was found independent of the applied magnetic field in the four foil flippers. The perfect spin echo is due to the fact that no magnetic aberration terms are included in the simulation.

### 3.1. SESANS with a scattering sample   

**Figure 7:**
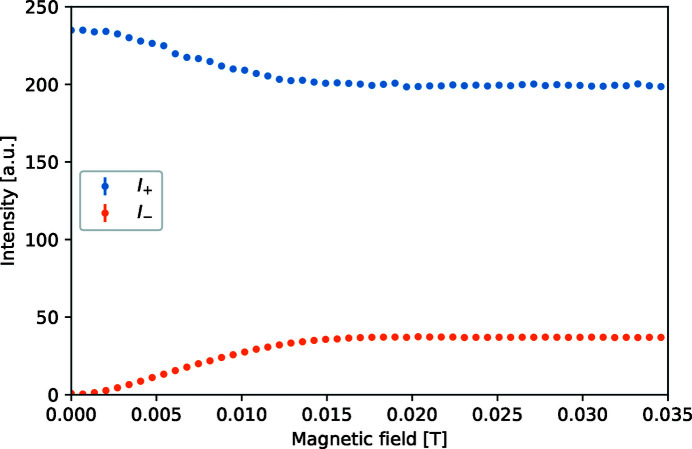
Detected intensity at the detector for the up and down orientations of the analyser as a function of the applied magnetic field in the foil flippers.

The complete instrument with sample is tested by scanning the magnetic field *B* in the four foil flippers and detecting the transmitted intensity with the analyser set in the plus or minus direction, *I*_+_ and *I*_−_. The raw simulation data are shown in Fig. 7 ▸.

**Figure 8:**
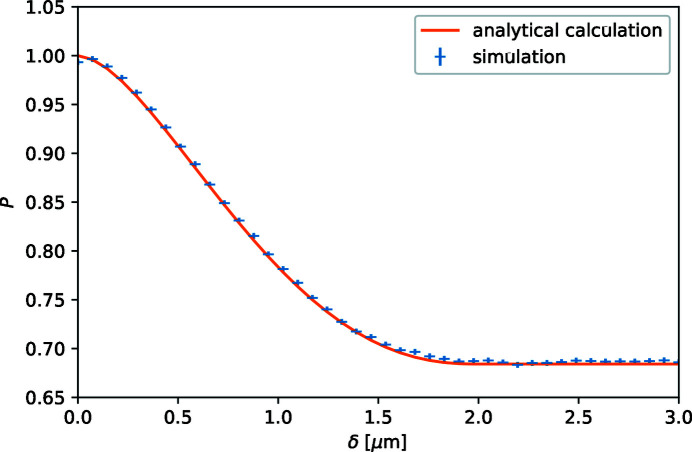
Calculated polarization from the simulated intensities in Fig. 7 ▸ as a function of spin-echo length in the SESANS setup after scattering by a sample of 1 µm radius spheres. The line is the theoretically calculated polarization curve for this sample.

From these intensities the polarization *P* can be calculated: 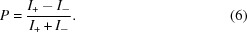
From the magnetic field the spin-echo length δ, corresponding to the probed length scale (Rekveldt *et al.*, 2005 ▸), can be calculated: 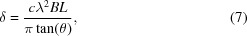
in which *L* = 1.5 m is the distance between the centres of the first and second foil flippers (which has to be identical to the distance between the third and fourth foil flippers). This polarization as a function of spin-echo length is plotted in Fig. 8 ▸ with a calculation of the analytical expected signal for the form factor of a solid sphere without a structure factor with the parameters for this sample according to the equations given by Krouglov *et al.* (2003 ▸). There is a perfect match between the simulation and calculation: the polarization starts at a value of unity and decays to a saturation value at a spin-echo length of 2 µm, corresponding to the diameter of the spheres, the longest length scale over which correlations in the sample are present. The polarization saturation value *P*(∞) = 0.68 at spin-echo lengths larger than 2*R* is in complete agreement with the expected value based on the earlier calculated scattering power τ: 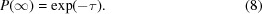
This indicates that this *McStas* SANS sample is scattering as it should do, including multiple scattering and the direct-beam properties.

## 4. SEMSANS   

### 4.1. Modulation   

**Figure 9:**
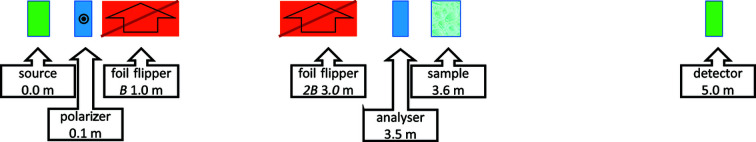
Schematic drawing of the *McStas* SEMSANS setup. Distances, sizes and angles are not to scale. The polarizer sets the neutron polarization out of the plane of the drawing. The magnetic field surrounding the second foil flipper is twice as strong as the corresponding field surrounding the first foil flipper.

The basic idea of SEMSANS (Fig. 9 ▸) is to create an intensity-modulated neutron beam at a position-sensitive detector (Gähler, 2006 ▸; Bouwman *et al.*, 2009 ▸). Any small-angle scattering by a sample in the beam will decrease the amplitude of this modulation (Bouwman *et al.*, 2011 ▸). Such a modulation can be achieved with two foil flippers for a certain focusing condition. The first foil flipper has a magnetic field *B*_1_ and is located at a distance *L*_1_ from the position-sensitive detector, while for the second foil flipper these parameters are *B*_2_ and *L*_2_, respectively. The focusing condition for a modulation is given by (Bouwman *et al.*, 2011 ▸)
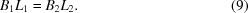
In the simulation the distances are set at *L*_1_ = 4.0 m and *L*_2_ = 2.0 m and the magnetic fields at *B*_2_ = 2*B*_1_ to fulfil this focusing condition.

**Figure 10:**
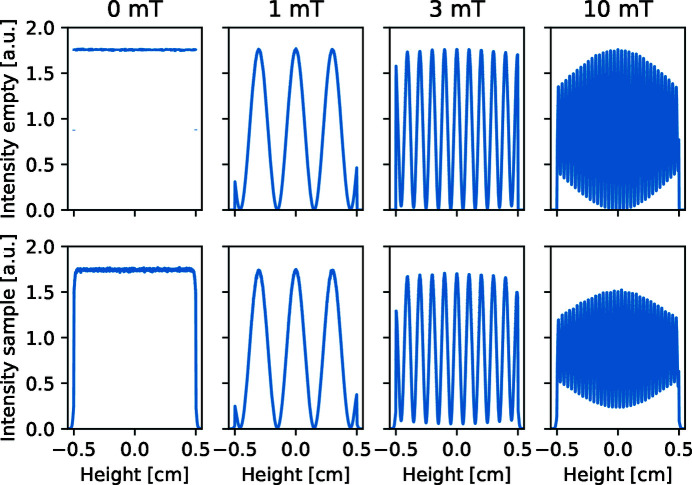
Graphs of simulation results of a SEMSANS instrument. In the top row is plotted the intensity at the position-sensitive detector as a function of height with different field strength. The field in the first magnet is given. The second row displays the intensity when a small-angle scattering sample with a radius of 1.0 µm is inserted after the second magnet.

In the top row of Fig. 10 ▸ the obtained modulation at the position-sensitive detector is presented for various settings of the magnetic fields in the foil flippers. The detector has 10018 × 1001 pixels, which gives sufficient resolution to observe even the modulations at the highest magnetic fields. The size of the detector is 11 × 11 mm, which allows us to intercept the full beam and observe any edge effects. The pixel size of 10 µm is just becoming achievable. The modulation has a smaller periodicity for higher magnetic fields, as expected. The modulation period ζ matches perfectly with the expected values (Bouwman *et al.*, 2011 ▸): 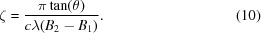
From the minimal and maximal intensities, *I*_min_ and *I*_max_, the ‘visibility’ *V* can be calculated:
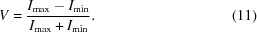
At field strengths of 1 mT in the first foil flipper magnet the intensity modulation is perfect, corresponding to a visibility of unity. For the field strength of 3 and 10 mT one can observe for the larger distances from the centre of the detector that the visibility of the modulation is decreasing. This decrease in visibility at the edges is due to the spread in wavelength (Δλ/λ = 0.01).

### 4.2. Modulation after a scattering sample   

Inserting a scattering sample after the second foil flipper will decrease the visibility when the modulation has a shorter period than the width of the scattering profile. The signal on the detector will be the original modulation convoluted with the scattering profile of the sample on top of the transmitted beam. The visibility of the modulation is thus the cosine transform of the scattering profile. This shows that the relative decrease of visibility with higher magnetic fields is completely equivalent to the formalism in SESANS (Bouwman *et al.*, 2011 ▸) and dark-field contrast imaging (Strobl *et al.*, 2015 ▸, 2016 ▸). The corresponding spin-echo length δ is described by 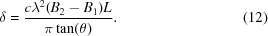
The four corresponding spin-echo lengths are thus 0.0, 0.14, 0.43 and 1.4 µm. The effect of these applied magnetic fields can be related to the change in polarization in the corresponding SESANS simulation in Fig. 8 ▸. That explains why scattering results in a decrease in modulation amplitude, only becoming visible at *B*_1_ > 3 mT. The effect is clearly visible by looking at the intensities at the minimums, which are no longer zero.

## 5. Conclusions   

*McStas* simulations of ideal SESANS and SEMSANS are in total agreement with analytical theories and experiments. The simulations clearly show how the polarization after one single foil flipper is completely lost and finally recovered again for the instruments without a sample. The most challenging part in these simulations is the correct description of the SANS sample, including the transmitted beam and the multiple scattering.

The principle of time-of-flight SEMSANS has been illustrated (Strobl, Tremsin *et al.*, 2012 ▸) and can now be simulated. Detection with a grating (Strobl, Wieder *et al.*, 2012 ▸) instead of a high-resolution detector is now also possible. This simulation development will make it possible to investigate more complex data-analysis challenges for combined SANS–SEMSANS measurements at time-of-flight instruments (Plomp *et al.*, 2007 ▸), as for example at the instrument Larmor at ISIS (Schmitt *et al.*, 2020 ▸). The scattering power and scattering angles are wavelength dependent, which gives interesting effects that require careful data reduction and that can now be simulated. Triangular precession coils as are used in some SESANS-like instruments (Pynn, Fitzsimmons *et al.*, 2008 ▸; Parnell *et al.*, 2015 ▸) are present in *McStas* (Sales, 2014 ▸), so for these instruments samples can now also be included in simulations.

In real instruments, imperfections (Uca *et al.*, 2003 ▸) and asymmetries in fields (Pynn, Lee *et al.*, 2008 ▸) put limits on the maximal fields that can be applied in precession devices before a loss of polarization in the spin-echo condition makes measurements impossible. Simulations will have to use realistic fields to find the imperfections that limit the technique and improve them. Imprecise manufacturing of components or misalignment’s can now be included in simulations to quantify their disturbance of the measurements. The simulations are a valuable tool for the further development of Larmor precession scattering methods.

In principle, one could adapt the sample to include a background and incoherent scattering to study their effect on the measurements. However, it will be more convenient and will give more insight to do such a study with analytical calculations. The modified SANS sample and the *McStas* instruments used in this study will be distributed with the forthcoming release of *McStas*.
